# Academy of Stomatology

**Published:** 1902-08

**Authors:** 

**Affiliations:** Academy of Stomatology


### Reports of Society Meetings.

### ACADEMY OF STOMATOLOGY.

A REGULAR meeting of the Academy of Stomatology of Philadelphia was held on the evening of Friday, December 27, 1901, at the rooms of the Academy, 1731 Chestnut Street, the President, Dr. S. B. Luckie, in the chair.

A paper entitled "The Burnished-Cap-Crown" was read by Dr. P. B. McCullough.

(For Dr. McCullough's paper, see page 545.)

### DISCUSSION.

*Dr. J. H. Gaskill.*—I should like to ask Dr. McCullough the advantage of making a first cap of platina and then fitting a gold band around that?

*Dr. McCullough.*—Of course, with a model of the prepared surface of the root in the laboratory the operator may form a cap after any manner he may fancy, but by making the cap after the manner represented here (indicating the incisor) there is no solder inside of the cap except where the dowel is soldered. The cap is deeper inside than upon its labial face, hence the least possible amount of gold shows. The porcelain is ground flat to the filed surface of the 22-carat gold band, thus insuring the most perfect joint with no soldered seam exposed, and in addition one has the contour of the natural tooth, a continuous curve from the cutting edge of the porcelain to the edge of the cap, while with every other form of cap the curve is interrupted at the point of union of the porcelain with the cap and usually requires the defacement of the porcelain. By virtue of the layer of solder between the turned edges of the cap and gold band it is stronger than any other form of cap.

The more perfect the imitation of the outline of the enamel, the less likely is gum irritation to occur.

*Dr. H. C. Register.*—I do not hesitate to say that there are as many, if not more, roots destroyed by crowning than are saved by it, because as of the production of irritation, which induces gingivitis leading to pericementitis and the other ills that follow irritation. The ferrule should be used with extreme care. I very rarely, except in exceptional cases, let it run up above the gum at all. I prefer a plate of gold or platinum well fitted to the face of the root. Through this is passed the post of a Logan crown, which has its labial edge nicely adapted to the labial part of the plate, while the lingual edge of the porcelain is well ground away, leaving a space between the crown and the plate. After being satisfied that everything is as correct as one can make it, simply catch it on the inside or outside with a quantity of phosphate of zinc. I use petroid for this. If using a gold plate, fill in the space with low-fusing porcelain body; bake it in the furnace, and it comes out a perfectly solid tooth with a little gold or platina termination, and the adaptation is exact. A plain plate tooth may be used if preferred. When in position on the root you have not got the ring or ferrule under the gum to act as an irritant. The festoon of the gingiva will become normal. The chief use of the ferrule is to prevent the root from splitting.

*Dr. S. H. Guilford.*—I would like to say a word in commendation of Dr. McCullough's method. I have looked over the specimens very carefully at his own office, and I have seen him do part of this work in his own laboratory. It has several novel features about it that are very excellent. There is one thing I liked, and that is its deviation from the common method of to-day,—namely, that of placing crowns, whether porcelain or hollow metal, in a hasty and careless manner. The band is a very useful thing, and I do not see how we can get along without it, but it does not necessarily create irritation. It simply needs to be properly made, to fit a root properly trimmed, and to be carefully adjusted. If that is done there is no more reason for irritation under or around the band than there would be without it. Of course, we do not pretend to make bands and force them beyond the free margin of the gum, but they are necessary. A ferrule is often necessary to avoid splitting, but that is not its only use. It prevents largely the washing out of the cementing substance which is apt to occur where no band is used, in spite of every precaution. One good feature of Dr. McCullough's method is that it is not too simple, hence not so apt to result in imperfect work. The pains taken in the several stages begets very accurate results, which should be encouraged because of the amount of quackery that has been brought about largely by the fact that crowns could be easily made and placed. The result has been careless and imperfect work. There are several features which he has explained that are really novel, and that I have not seen anywhere else.

It is all important to have as little space between the crown and root as possible. The main dependence should be the matter of fit. The cementing material should be placed to keep out the fluids of the mouth rather than for the purpose of holding the crown in position. A plate attached as he suggests would solve the problem of removal for cleansing.

*Dr. J. H. Gaskill.*—To my mind the only trouble with a ferrule crown is the badly fitting ferrule due to an improperly prepared root. The objection which I have to this crown of Dr. McCullough's is the width of band above the porcelain facing. Dr. D. D. Smith, I think, first suggested the plan of cutting well away from the labial or buccal portions of the root; so that the facing could be carried to the edge of the ferrule, thereby obviating a possibility of the gold band showing, as it is entirely covered by the porcelain.

The method of making such a crown is to make a band fit the root, which has been prepared as before described; cut out the front portion and grind the facing so that the band is covered clear to the edge; this is then soldered to the band. The cusps are then shaped up and soldered to the band and facing. The pin is cemented in the root independent of the crown, the cement in the crown holding all together.

Should a facing break off, it can easily be replaced by drilling two holes in the position of the old pins, a facing ground to fit, and the pins spurred and fastened in place with cement; this repair will be as strong as the original facing.

*Dr. Guilford.*—I would like to say that the objection to the crown that Dr. Gaskill illustrates is that it requires a large amount of cement in the setting. It has certain advantages of first anchoring the dowel in the root and then putting the crown into it. But it involves filling up the large space with cement.

*Dr. Gaskill.*—How about an all-gold crown?

*Dr. Guilford.*—There is the same objection. With an all-gold crown it is well to allow but little space between the gold crown and the tooth, and then set with gutta-percha. The use of large masses of zinc phosphate is objectionable, because they take up the fluids of the mouth and become foul. Recently I removed a couple of crowns that were placed several years ago, in the manner Dr. Gaskill describes, and they were the most offensive things I ever saw. Where cement is used it should be used on the root first and properly shaped, so that when the crown goes over it it will require a less amount of cementing substance to hold it. The crown or bridge can be taken off at any time as well.

I use the red base plate. Line the crown with a coating of gutta-percha in solution. Heat the base plate and stretch it out until thin and cut into narrow strips and line the crown with it; the chloropercha will attach it. Heat the crown and try upon the wet tooth, trim, heat again, and reapply, and so on until it almost goes into place. The root is then thoroughly dried and coated with oil of eucalyptus or cajeput, which will dissolve the gutta-percha, and the heated crown is to be pressed into place, a napkin being used to hold it. When placed it can never be gotten off without heat. Where the crown is banded like certain of these, coat the pins or dowel with a solution of gutta-percha, place a thin strip of gutta-percha on the inside of the band, then proceed as before. The old way of heating the crown was objectionable, as so much heat was used that it burned the gum; by this method the gum is not injured. The crown may be taken off by using heat to soften the gutta-percha, but not in any other way.

*Dr. F. A. Peso.*—There seems to be a great deal of merit in these methods, but I have had no practical experience with them. All depends upon the manner in which the work is done, and thoroughness in everything makes good work.

*Dr. E. C. Rice.*—One of the best points in this work is that in setting these crowns it is impossible to force the crown so far into the gum as to cause injury. The ferrule first having been made of the proper depth and the cap soldered on, the crown is put in place before the cement is put into the ferrule. It reaches the exact point of occlusion, and when the cement has been put into the ferrule and the crown forced into place it cannot go down any farther than when it was first tried on, no matter how much pressure is put upon it.

*Dr. H. E. Roberts.*—I feel like congratulating Dr. McCullough on the careful and workmanlike way in which he has prepared the specimens shown on the cards. The objection that Dr. Register has made to band crowns or ferrule crowns could certainly be obviated by Dr. McCullough's method, as he cannot force it down any distance under the gum. I have seen a number of ferrule crowns and all-gold crowns which are open to the objections that Dr. Register makes, and I cannot condemn them sufficiently. When a man will make a gold crown and use a mallet to force it up, never minding the patient's feeling, until he gets up to the process or above it, you can certainly expect to have trouble and lose teeth.

*Dr. C. R. Jeffries.*—I have not made crowns following these methods. I can admire the ingenious manner in which it has been conceived. In the battle with teeth, the success or failure, the excellence or the opposite, depends upon the men who do the work.

*Dr. McCullough.*—The idea of the V-shaped space cut in the upper bicuspid crown to set the porcelain, is that the thicker the facing, the less gold to be used. By the porcelain running back, it will not shade dark between the teeth in the mouth. By filing these lines perfectly flat, and by grinding both ends of the facing flat, you have with least labor the most perfect joint. As there is very little change in the soldering and very little space to be filled, and the tendency is to draw the porcelain tighter, there is no change in the joints between the gold and porcelain, as is apt to occur in the present method of making bicuspid crowns. You will observe that with many crown systems the merits are summed up with the objectionable features in the detail of construction left out. It is believed that there is no system of crowning in which a result is obtained, or even approaching it, with so little pain on the part of the patient and with the accuracy of results, as this. For example, with every system advertised, the manufacturer avers that there is perfect articulation. Now, none of them provide a method for getting the action of the jaws, consequently they only have occlusion and never have articulation. They say they have perfect contour. The ready-made crowns that I have seen are bevelled on the palatine and buccal surface, and others that I have seen form the same curve on the palatal and buccal surfaces, hence the impossibility of contour.

Now, whether the band is fitted directly to the root or the measure taken with wire, there is no provision by any of these methods for fitting a fissure in a root that may extend down to the point where it is ground off below the gum. By taking this impression of the properly prepared root, the bevel is reproduced on the model to which the cap can be accurately fitted.

In one case crowned after this system the upper molar was so badly decayed as to totally obliterate the pulp cavity; considerable gum had to be cut away, and the crown anchored by pins being set independently in the roots. At the present time (after two years) the relation between the gum and crown is absolutely natural in appearance. In cases where it has been necessary to cut away process to get bevel, the gum has without exception become normal. In cases where a slight gingival irritation is present upon the lingual surface of several teeth, with a perfectly healthy condition buccally, the same condition has appeared upon the crowned teeth. The first visit is for the preparation of the root and taking the impression, the second for fitting the dowel or taking the bite, and the third visit to set the finished crown. In cases where there are no occluding teeth the molar crowns may be set at the second visit of the patient. In the case of split teeth, it usually happens that the split section slants off under the gum. In such cases the root may be prepared in two ways for the impression. The sections may be ligated, the root bevelled, an impression taken, the split section removed and marked in the impression; or the split off piece may be taken away and the impression tube cut so as to include the lines of fracture. It seems to be the only system that applies alike to every tooth in the mouth in any condition. The conception of taking an impression and forming a cement model cannot help being of advantage, as it makes it possible for work to be done in the laboratory heretofore done in the mouth. By making the cap for the molar crown it serves as a guide in setting and reinforces the crown at the edge fitting the root. The detail of this system provides for fit contour, approximal contact, articulation in contradistinction to occlusion, and parallel alignment. Natural teeth may be set in the mandrel section of the swaging device and any surface reproduced by swaging. Crowns for regulating appliances may be made by filling the plaster impression of the occlusal surface of the tooth to be crowned with zinc phosphate, and upon this swaging the cusps, which when soldered to the band fitted around the plaster tooth forms a crown that may be set at the second visit of the patient.

Adjourned.

OTTO E. INGLIS,
*Editor Academy of Stomatology.*

